# Coccidioidal Peritonitis: Clinical Characteristics, Treatment and Outcomes

**DOI:** 10.3390/jof12070489

**Published:** 2026-07-03

**Authors:** Jamilah L. Shubeilat, Sandhya R. Nagarakanti, Abeer Almajali, Jaxon K. Quillen, Matthew R. Buras, Lisa Speiser, Janis Blair

**Affiliations:** 1 Division of Infectious Diseases, Mayo Clinic Arizona, Phoenix, AZ 85045, USA; 2 Department of Quantitative Health Services, Division of Clinical Trials and Biostatistics, Mayo Clinic—Samuel C. Johnson Research Center, Scottsdale, AZ 85059, USA

**Keywords:** peritoneal, peritonitis, coccidioidomycosis, *Coccidioides*, antifungal treatment, disseminated infection

## Abstract

Peritoneal coccidioidomycosis is a rare, disseminated manifestation of *Coccidioides* infection with limited representation in the literature. We report 37 proven or probable cases from a single institution, including 12 patients (32%) with immunosuppression. Thirty-six patients received antifungal therapy, all of whom demonstrated clinical response. Among 11 (29.7%) patients who completed and stopped therapy after a median duration of 26 months, 2 (5%) experienced a relapse and were successfully retreated. Nine patients (24%) had no known relapse. Thirteen patients (35%) required modification of antifungal regimens, primarily due to azole adverse effects. Treatment approaches and median duration of therapy (57 months) were similar between immunosuppressed and non-immunosuppressed groups. No deaths were attributable to infection. Overall, outcomes were favorable, with strong clinical responses to azole therapy. Patients without identifiable immunologic deficits or additional sites of dissemination, when managed with antifungal therapy and close clinical, radiologic, and serologic monitoring, may achieve good prognosis, particularly when indefinite azole therapy is not feasible.

## 1. Introduction

Coccidioidomycosis (CM), also known as Valley Fever (VF), is an infection caused by the dimorphic pathogenic fungi *Coccidioides immitis* and *Coccidioides posadasii* [1]. This infection is endemic to the southwestern United States as well as parts of Central and South America [1]. The disease is acquired via inhalation and primarily presents as a respiratory infection, which is frequently mistaken for bacterial or viral community-acquired pneumonia (CAP) [2]. Extrapulmonary CM occurs in approximately 1–5% of patients [3]. The infection can spread to almost any part of the body, commonly the soft tissues, bones, joints, and meninges [2]. Peritoneal coccidioidomycosis (PC) is a rare but observed manifestation of the disease, and limited data has been published about its optimal management and the disease course. In this article, we review 37 cases of PC, seen and managed at a single institution.

## 2. Materials and Methods

This study was approved by the Institutional Review Board (IRB) of Mayo Clinic. We used International Classification of Diseases, Ninth and Tenth revisions (ICD-9, ICD-10, respectively) codes to search electronic medical records. Specifically, the Mayo Data Explorer, a self-service data exploration and retrieval tool, was used to search electronic records for the following ICD-9 and ICD-10 codes: disseminated coccidioidomycosis, other forms of progressive coccidioidomycosis, and primary extrapulmonary coccidioidomycosis. We reviewed the electronic medical records of all patients identified to find all patients with a diagnosis of PC. Cases with inadequate data were excluded. Cases of coccidioidal intraabdominal abscesses without clear evidence of a generalized peritoneal inflammatory process were excluded. Cases of secondary PC complicating ventriculoperitoneal (VP) shunts were included. Proven cases were defined by positive peritoneal or ascitic fluid cultures or with a finding of spherules consistent with *Coccidioides* in cytology fluid or tissue on pathology. Probable cases were defined based on clinical presentation, positive serology, and ascitic fluid analyses. Infectious disease notes and relevant clinical data, including imaging studies and CM serological results, were reviewed from the time patients initiated care through December 2025. The end of therapy was defined as the date on which the treating clinician elected to discontinue treatment during follow up. Relapses were determined by treating physicians based on clinical assessment and supporting serological and/or radiological findings. Patient charts were manually reviewed and pertinent clinical, radiological and laboratory variables were collected.

## 3. Statistical Analysis

Categorical variables are summarized as count (percent) while numerical variables are summarized as mean (standard deviation) as well as median and interquartile range, represented by the first and third quartile (Q1, Q3), and range (minimum–maximum). All analyses were performed in R 4.4.1. [4].

## 4. Results

From 1 January 2005 to 31 December 2025, we identified 945 potential patients, 37 of whom met case definitions of proven (*n* = 28) and probable (*n* = 9) peritoneal coccidioidomycosis. Cases were identified in the inclusive years ranging from 2006 to 2025. Table 1 summarizes patient characteristics, and Supplemental Table S1 provides details of patient characteristics. The median age was 50 years (range 22–81). Among 30 patients with available data, the median duration of symptoms prior to diagnosis was 4 months (range of 0–120 months). Fourteen patients were immunosuppressed. Two patients had diabetes mellitus based on a HBA1 of 6.5 or higher, and six had prediabetes HBA1c ranges between 5.7 and 6.4%.

Supplemental Table S2 details clinical symptoms, radiological findings, serology data, cultures, and histopathology data. Diagnostic features are further summarized in Table 1. Twenty-six (70.3%) patients presented with abdominal symptoms (nausea, vomiting, pain, distention, and ascites). Other symptoms included fatigue, fever, cough, weight loss, infertility, skin findings, and headaches. Thirty-two patients (86.5%) had evidence of pulmonary involvement before or at the time of diagnosis. Peritoneal coccidioidomycosis was the primary presentation in 59.5% of patients (22/37). Seventeen patients (46%) had other extrapulmonary involvement beyond the peritoneum, which included the central nervous system (CNS), abdomen and pelvis, lymph nodes, and skin. Serologic data was available in 36 patients, and all were seropositive. Complement fixation titers ranged between 1:4 and 1:1024, with a median titer of 1:128. Imaging data was available in 34 patients, and all had abnormal findings on CT, MRI, ultrasound, or PET-CT scan. Fifteen patients had diagnostic paracentesis. Supplemental Table S3 details ascitic fluid results for patients with available data (*n* = 15).

Antifungal therapies used, duration of therapy, and outcome of therapy are detailed in Supplemental Table S4. A summary of treatments by immunocompromised status and by deceased status are respectively summarized in Table 2 and Table 3. Thirty-six patients (97.3%) received antifungal therapy for 5–129 months (median 57). One patient was diagnosed based on findings at the time of hernia surgery, was observed off-therapy, and did well. A patient who had a VP shunt in place was treated without removal of the shunt. Antifungals used were liposomal amphotericin, fluconazole, posaconazole, voriconazole, itraconazole and isavuconazole. Patients with immunosuppression (IS) were treated with similar antifungals when compared with those without IS. The median duration of therapy was 57 months in both groups. Five patients died while on treatment, but none were attributed to disseminated coccidioidomycosis. We reviewed clinical notes including discharge summaries to determine cause of death. Death was attributed to progressive cardiopulmonary and renal dysfunction in two patients. One patient died secondary to active variceal bleeding. We could not determine the cause of death for the remaining patients as there were no available data.

Eleven patients completed and stopped antifungal therapy. Two of these 11 (18%) relapsed during follow up; one with T-cell lymphopenia/lymphoma relapsed after 24 months off antifungal treatment and presented with further disseminated CM. The second patient, without IS, had an end of treatment titer of 1:2 which rose to 1:128 after 6 months of observation; both patients were retreated. Nine other patients who completed and remained off therapy did not experience relapsed infection.

Supplementary Table S5 details the outcome of patients who stopped/completed therapy during the follow-up period without documented evidence of relapse during the follow-up period (*n* = 9), which were often characterized by non-adherence due to adverse effects.

Thirteen patients required a change in antifungal regimen due to either side effects, clinical failure, or financial reasons. The most common reason for changing therapy was side effects (10/13, 76.9%). Among the 37, 6 patients died during the follow-up period. No mortality was attributed to PC. Patients who died had received SOT (*n* = 3) or had cirrhosis (*n* = 2) or T-cell lymphopenia/lymphoma (*n* = 1).

## 5. Discussion

The incidence of extrapulmonary coccidioidomycosis varies among different populations, which can be as low as 0.5% for patients of Caucasian origin to multiple folds higher in patients of African or Filipino origin [5]. The incidence is also significantly higher, approaching 50% of cases, in immunocompromised individuals, especially those with impaired cellular immunity, such as those with uncontrolled HIV infections, patients with hematological malignancies, SOT, or patients receiving high-dose corticosteroids or anti-tumor necrosis factor (anti-TNF) medications for autoimmune diseases [5].

Peritonitis remains an uncommon manifestation of coccidioidomycosis. PC presents with vague nonspecific abdominal symptoms and subclinical ascites and therefore can be missed with a delay in diagnosis [6]. Twenty-three cases were reported by multiple authors from multiple medical centers in 1965 [7]. Aside from meningeal infection, where the disease is almost always fatal if left untreated, the outcome of extrapulmonary nonmeningeal coccidioidomycosis is widely variable [7]. Determining how to best manage peritoneal coccidioidomycosis is not known, and published information is limited to a few case reports and a multicenter series of 17 cases from California [6]. Even with the recent publication of 17 cases, optimal treatment was not known.

The current series of 37 cases represents the largest published cohort of peritoneal coccidioidomycosis to date. Our study highlighted the potentially subtle presentation of PC and the crucial need to keep high suspicion for the disease to allow early diagnosis and management. Incidental histopathological findings of PC were observed in our case series in patients presenting with infertility, endometriosis, and while presenting for surgical intervention for an alternative diagnosis. Typically, for the patients with ongoing cellular immunity impairment, we have recommended continued suppressive antifungal treatment. The approach for the non-immunosuppressed has been more difficult in the absence of data. The most common reason patients stopped their antifungal therapy in our series was intolerance of medication due to side effects. As a result, we identified a cohort of patients (non-immunosuppressed, without other extrapulmonary coccidioidal sites) who may be eligible to antifungal treatment discontinuation and close follow up to monitor for possible relapsed infection.

The Infectious Diseases Society of America coccidioidomycosis treatment guidelines recommend “other soft tissue” sites, such as peritonitis, to be treated similar to cutaneous and subcutaneous abscesses [8]. Oral azoles are the antifungals of choice. Amphotericin-B is sometimes used to initiate treatment in severe or rapidly progressing disseminated infections [8]. Most patients in our series did not require amphotericin-B. Response rates of soft tissue nonmeningeal CM vary, and relapse rates decrease, approaching 11% after 12 months of therapy [9]. Given the high relapse rates, society guidelines recommend at least 6–12 months of therapy [8]. Many experts treat for 12–24 months and only consider discontinuation of treatment after close clinical, laboratory and radiological monitoring and after careful discussion with patients and counseling about the risk of relapse of CM. The median duration of treatment in our case series was 57 months.

Intact cellular immunity is crucial for controlling CM and interferon-γ-mediated response; type 1 immune response leads to infection resolution [10]. Type 2 immunity predominates over type 1 immunity in conditions linked to disseminated infection including pregnancy, HIV, and acquired immunodeficiency syndromes including immunosuppression with steroids or biologics, but we have yet to completely understand the full immunological response leading to CM dissemination [10]. The current case series suggests that perhaps patients with PC have a more favorable response than other disseminated coccidioidal infection. However, more patients and a longer follow-up time are required prior to making further conclusions.

The current study has many acknowledged limitations. This study was conducted at a tertiary referral center, with a typically immunosuppressed population, and a racial distribution that does not reflect the general population. Therefore, our experience may not be generalizable to other populations. There was no standardized treatment algorithm used, and multiple physicians were involved in treatment decisions. For some otherwise immunologically normal patients, discontinuation of treatment was not considered, while other patients’ total time on treatment was influenced by ongoing adverse effects of treatment and patient adherence.

## 6. Conclusions

Clinicians must consider peritoneal coccidioidomycosis in patients presenting with abdominal symptoms, ascites, peritonitis or evidence of pathological granulomatous inflammation in areas endemic for coccidioidomycosis, in particular while presenting with pulmonary manifestations. Our experience suggests that a carefully monitored specific subgroup of patients may have a favorable prognosis with discontinuation of antifungal therapy with close clinical and serological monitoring every 3–6 months and radiological follow up with a frequency that can be determined based on patients’ initial radiological findings. Further study of affected patients is required to provide guideline level recommendations for treatment of this unusual infection.

## Figures and Tables

**Table 1:** Baseline clinical characteristics and demographics.

	Overall (*N* = 37)
Age (at Diagnosis)	
N	37
Median (Q1, Q3)	50.0 (41.0, 61.0)
Mean (SD)	50.0 (13.8)
Range	22.0–81.0
Gender	
N	37
Male	19 (51.4%)
Female	18 (48.6%)
Ethnicity/Race	
N	37
American Indian/Alaskan Native	1 (2.7%)
Asian	4 (10.8%)
Black	4 (10.8%)
Hispanic or Latino	3 (8.1%)
White	24 (64.9%)
Not Disclosed	1 (2.7%)
Initial Symptoms: Abdominal Symptoms &/or Ascites	
N	37
No	11 (29.7%)
Yes	26 (70.3%)
Infertility	
N	37
No	36 (97.3%)
Yes	1 (2.7%)
Had Antifungal Therapy	
N	37
No	1 (2.7%)
Yes	36 (97.3%)
Duration of Antifungal Therapy (in months)	
N	31
Missing	6
Interrupted	1 (3.2%)
<6 Months	1 (3.2%)
6–12 Months	1 (3.2%)
1–3 Years	5 (16.1%)
3–5 Years	0 (0.0%)
>5 Years	2 (6.5%)
Remains on Therapy/Indefinite	21 (67.7%)
Duration of Antifungal Therapy by 31 December 2025 (in months)	
N	23
Missing	14
Median (Q1, Q3)	58.0 (43.5, 101.0)
Mean (SD)	67.3 (40.3)
Range	5.0–142.0
Was the Mortality Related to Cocci?	
N	4
Missing	33
No	3 (75.0%)
Yes	0 (0.0%)
Unknown-Patient passed away suddenly in his home	1 (25.0%)
Hemoglobin A1c at diagnosis or first available after diagnosis	
N	22
Missing	15
Median (Q1, Q3)	5.4 (5.0, 6.0)
Mean (SD)	5.6 (0.7)
Range	4.8–7.4
Is patient SOT?	
N	37
No	31 (83.8%)
Yes	6 (16.2%)
On Immunosuppressants?	
N	37
No	25 (67.6%)
Yes	12 (32.4%)
Duration of Symptoms Prior to Dx (In Months)	
N	30
Missing	7
Median (Q1, Q3)	4.0 (2.0, 6.0)
Mean (SD)	10.2 (23.4)
Range	0.0–120.0
Any Pulmonary Involvement?	
N	37
No	5 (13.5%)
Yes	32 (86.5%)
Was it Primary Presentation?	
N	37
No	15 (40.5%)
Yes	22 (59.5%)
PC Serology	
N	36
Missing	1
Positive	36 (100.0%)
Titer	
N	33
Missing	4
Median (Q1, Q3)	128.0 (32.0, 256.0)
Mean (SD)	199.4 (252.7)
Range	4.0–1024.0
Extra Pulmonary Site	
N	37
No	20 (54.1%)
Yes	17 (45.9%)
MIC: Positive Peritoneal Culture?	
N	32
Missing	5
No	13 (40.6%)
Yes	12 (37.5%)
Not Available	7 (21.9%)
Positive Peritoneal Histopathology?	
N	37
No	11 (29.7%)
Yes	16 (43.2%)
Not available	10 (27.0%)
Imaging Results	
N	37
Abnormal Findings	34 (91.9%)
Not Available	3 (8.1%)
Deceased While on Treatment	
N	37
Deceased not while on Treatment	1 (2.7%)
Deceased while on Treatment	5 (13.5%)
Not Deceased	31 (83.8%)

Abbreviations: N, number; Q1, Q3, first and third quartile, respectively; SD, standard deviation.

**Table 2:** Treatment and outcomes by immunosuppressant use.

	No (*N* = 25)	Yes (*N* = 12)	*p* Value
**Duration of Antifungal Therapy (in months)**			
N	20	11	
Missing	5	1	
Interrupted	0 (0.0%)	1 (9.1%)	
<6 Months	1 (5.0%)	0 (0.0%)	
6–12 Months	1 (5.0%)	0 (0.0%)	
1–3 Years	5 (25.0%)	0 (0.0%)	
3–5 Years	0 (0.0%)	0 (0.0%)	
>5 Years	1 (5.0%)	1 (9.1%)	
Remains on Therapy/Indefinite	12 (60.0%)	9 (81.8%)	
**Therapy Completed?**			0.214 ^1^
N	20	11	
Missing	5	1	
No	12 (60.0%)	9 (81.8%)	
Yes	8 (40.0%)	2 (18.2%)	
**Relapse**			0.585 ^1^
N	25	12	
No	24 (96.0%)	11 (91.7%)	
Yes	1 (4.0%)	1 (8.3%)	

^1^ Pearson’s Chi-squared test. Abbreviations: N, number.

**Table 3:** Treatment by deceased status.

	No (*N* = 31)	Yes (*N* = 6)
**Duration of Antifungal Therapy (in months)**		
N	29	2
Missing	2	4
Interrupted	0 (0.0%)	1 (50.0%)
<6 Months	1 (3.4%)	0 (0.0%)
6–12 Months	1 (3.4%)	0 (0.0%)
1–3 Years	5 (17.2%)	0 (0.0%)
3–5 Years	0 (0.0%)	0 (0.0%)
>5 Years	2 (6.9%)	0 (0.0%)
Remains on Therapy/Indefinite	20 (69.0%)	1 (50.0%)
**Duration of Antifungal Therapy by 31 December 2025 (in months)**		
N	20	3
Missing	11	3
Median (Q1, Q3)	54.5 (40.0, 91.5)	117.0 (101.5, 129.5)
Mean (SD)	60.1 (37.2)	115.0 (28.1)
Range	5.0–129.0	86.0–142.0
**Therapy Completed?**		
N	29	2
Missing	2	4
No	20 (69.0%)	1 (50.0%)
Yes	9 (31.0%)	1 (50.0%)

Abbreviations: N, number; Q1, Q3, first and third quartile, respectively; SD, standard deviation.

## Data Availability

No new data were created or analyzed in this study. Data sharing is not applicable to this article.

## Supplementary Materials

The following supporting information can be downloaded at https://www.mdpi.com/article/10.3390/jof12070489/s1, Table S1: Demographic and Underlying Medical Characteristics. Table S2: Patient initial symptoms, radiological findings, serology data, cultures and histopathology data. Table S3: Peritoneal fluid analyses for patients with available data (*n* = 15). Table S4: Antifungal therapy, duration of therapy. Table S5: Outcome of patients (*n* = 9) who stopped/completed therapy during follow up period.

## Abbreviations

CAP	community-acquired pneumonia
CM	coccidioidomycosis
ICD	International Classification of Diseases
PC	peritoneal coccidioidomycosis
VF	valley fever
