# Supplementary material for: Coccidioidal Peritonitis: Clinical Characteristics, Treatment and Outcomes
Source: J Fungi (Basel). 2026 Jul 3;12(7):489. doi: 10.3390/jof12070489 (PMC13412693; doi:10.3390/jof12070489)
Supplement: Supplementary file 1 [file jof-12-00489-s001.zip › jof-4325825-supplementary.pdf]

Supplemental Table S1. Demographic and Underlying Medical Characteristics

Patient Number	Sex	HBA1C	HIV status	SOT	IS
1	M	4.9	Negative	Yes	Yes – adalimumab, etanercept & SOT
2	M	5.9	Negative	No	No
3	F	5	Negative	Yes	Yes - SOT
4	F	NA	Negative	No	No
5	F	NA	NA	No	Yes - Abatacept
6	F	5.6	NA	No	Yes - Infliximab
7	M	4.8	Negative	Yes	Yes - SOT
8	F	5.2	Negative	No	No
9	M	6.3	Negative	No	No
10	F	5.4	Negative	No	No
11	M	6	Negative	No	No
12	F	5	Negative	No	No
13	F	5.2	Negative	No	Yes - Prednisone
14	M	NA	NA	No	No
15	F	NA	Negative	No	No
16	F	4.8	Negative	No	No
17	M	5.4	Negative	No	No
18	M	NA	Negative	No	Yes - Infliximab
19	F	NA	Negative	No	No
20	M	NA	Negative	No	No
21	M	7.4	Negative	No	Yes - Infliximab
22	F	NA	Negative	No	No
23	M	NA	Negative	No	No
24	M	6	Negative	No	T-cell lymphopenia, Lymphoma
25	M	6.3	Negative	No	No
26	M	NA	NA	Yes	SOT
27	M	5.6	Negative	No	No
28	F	NA	Negative	No	No
29	F	5	Negative	No	No
30	M	6.9	Negative	Yes (Liver)	Yes -Prednisone, Everolimus
31	M	NA	Negative	No	No
32	M	4.9	NA	No	No
33	F	NA	NA	No	No
34	F	NA	NA	No	No
35	F	5.3	NA	Yes (Kidney)	Tapered off steroids prior to diagnosis of DC
36	F	NA	NA	No	No

37	M	5.8	No	Yes- Received kidney after diagnosis	No at the time of diagnosis and treatment

Abbreviations:
AZ, Arizona
DC, disseminated coccidioidomycosis
F, female
FL, Florida
IS, immunosuppression
M, male
NA, data not available
PC, peritoneal coccidioidomycosis
SOT, solid organ transplant

Supplemental Table S2. **Patient initial symptoms, radiological findings, serology data, cultures and histopathology data**

Patient Number	Initial symptoms	Image findings	cocci serology data at dx	positive peritoneal culture?	positive peritoneal histopathology
1	Fever, chills, sweats, cough	MR abdomen no ascites or fluid collection	Positive EIA, ID, CF (1:32)	Not available	Yes
2	Abdominal pain, constipation	CT Abd/pelvis ascites, dilated bowel loops	Positive EIA, ID, CF (1:64)	Not available	Yes
3	Nausea, vomiting, Fever	CT abdomen pyelonephritis of transplanted kidney	Positive EIA, ID, CF (1:256)	Yes (peritoneal lymph node)	Yes
4	Abdominal pain, bloating	Ascites, inflammatory changes in the mesentery	Positive EIA, ID, CF (1:256)	Not available	No
5	Rash, Fatigue, anorexia, weight gain	Moderate Free intraperitoneal fluid, abnormal peritoneal densities	Positive CF (1:64)	Not available	Not available
6	Fatigue, Abdominal pain	Not available	positive CF (1:32)	Not available	Not available
7	abdominal pain, diarrhea, abdominal	Diffuse thick-walled peritoneum, moderate ascites, omental stranding and nodularity	Positive cocci EIA, ID, CF (1:256)	Yes	Yes

	distention, cough				
8	Abdominal bloating	Left ovarian mass, omental nodules and colonic thickening	Positive cocci EIA, ID, CF (1:128)	Not available	Yes
9	Recurrent ascites 2/2 cirrhosis	Cirrhotic liver, chronic portal vein occlusion, moderately severe portal hypertension with large varices	Positive EIA IgG, ID IgG, CF (1:256)	Yes	No
10	Abdominal bloating and distention	Ascites	Positive cocci EIA, ID, CF (1:128)	No	No
11	Abdominal bloating abdominal pain	New onset ascites, omental edema and omental nodularity	Positive cocci EIA, ID, CF (1:1024)	No	No
12	Abdominal bloating, change in BM, endometriosis vs IBS	Ascites and omental induration	Positive EIA IgG, ID IgG, CF (1:256)	Yes	Yes
13	Abdominal symptoms	Fungal ball and intraabdominal abscesses at the VP shunt	Positive cocci EIA, ID, CF (1:8)	Yes	No
14	No symptoms (left hernial repair)	nonspecific fluid stranding and infiltration of the omentum and mesenteric fat	Positive cocci EIA, ID, Negative CF	No	Yes
15	Abdominal pain, abnormal uterine bleeding	diffuse peritoneal and omental nodular thickening	Positive cocci EIA, ID, CF (1:8)	No	Yes
16	Abdominal pain and swelling	Ascites and mesenteric edema	Positive cocci EIA, ID, CF (1:16)	Yes	No
17	Abdominal swelling	possible cirrhosis with borderline splenomegaly and moderate ascites	Positive cocci EIA, ID, CF (1:512)	Not available	Not available
18	Abdominal pain, joint pain, fatigue	Ascites, multifocal lymphadenopathy, hepatic, adrenal & splenic lesions	Positive cocci EIA, ID, CF (1:256)	No	No

19	Abdominal bloating, pain and distention	Ascites, Omental nodularity	Positive cocci EIA, ID, CF (1:256)	No	Yes
20	Cough and abdominal pain	Peritoneal nodularity and thickening	Positive cocci EIA, ID, CF (1:256)	Yes	No
21	abdominal pain	small-to-moderate volume ascites and mesenteric edema	Positive cocci EIA, ID, CF (1:8)	Yes	Yes
22	Infertility	peritoneal nodularity	Positive cocci EIA, ID, CF (1:4)	No	Yes
23	Abdominal pain, bloating	Ascites, peritoneal nodularity	Positive cocci EIA, ID, CF (1:1024)	No	No
24	Ascites	Diffuse omental and peritoneal carcinomatosis of unknown primary, multiple prominent periaortic lymph nodes, bilateral adrenal glands enlargement	Positive cocci EIA, ID, CF (1:128)	Yes	NA
25	abdominal pain, ascites	Not available	Positive cocci EIA, ID, CF (1:64)	NA	Yes
26	Diagnosis at time of liver transplant	Hepatic cirrhosis features with HCC	Not available	NA	Yes (fibrosis and sclerotic granulomas containing spherules)
27	chronic illness, weight loss, fatigue	Abnormal uptake in abdomen on PET	Positive cocci EIA	N/A	No (multifocal fibrosclerosis)
28	Abdominal bloating and distention	Not available	Positive cocci EIA, ID, CF (1:8)	NA	Yes
29	Abdominal pain, drainage	Extensive subcutaneous abscesses in left upper and lower abdominal & pelvic, multiple sinus tracts to the skin surfacer studies. Extension to	Positive Cocci EIA, ID, CF (1:512)	Yes- abdominal swab cultures	NA

		rectus abdominis muscle, external oblique muscle.<br><br>Lymphadenitis			
30	Lesion over Chin	US abdomen- large volume ascites: CT chest: Diffuse miliary nodularity, septal thickening and larger upper lung cavitary nodules.	Positive Cocci EIA, ID, CF (1:64)	No	NA
31	Abdominal bloating, ascites, shortness of breath, diarrhea, pleuritic chest pain	CT abdomen- Ascites, CT chest-CT showed large low attenuation, somewhat necrotic-appearing mediastinal and hilar nodes, with focal air space nodular infiltrate in the medial basilar segment of the right lower lobe, that appears to invade the mediastinum with no discrete fat plane between the infiltrate and esophagus	Positive Cocci Screen, ID, CF (1:256)	Yes- peritoneal cultures	NA
32	Rib pain, Abd pain, fever, night sweats	CT scan showed an enlarged spleen, omental mass, and retroperitoneal lymph nodes	Positive Cocci IgG EIA, ID, CF (1:64)	no	NA
33	Fatigue, tiredness	CT of the chest and abdomen had revealed ascites as well as peritoneal thickening and a left upper lobe nodule with some inflammation	CF 1:32	No	Yes-Peritoneal biopsy with spherules
34	Flu like symptoms with fevers, chills, and shortness of breath, ankle	CT abd : adnexal masses and ascites. CT chest May 2022: 4 and 5 mm posterior RUL noncalcified nodules, 3 mm	CF 1:256	No	laparoscopic biopsy of peritoneal mass and abdomen which revealed granulomatous

	swelling, abdominal pain, swelling	noncalcified nodules in RML.			disease with fungal organisms consistent with cocci.
35	Chills, Fatigue, body aches, arthralgia, weight loss	3.3 cm hilar mass of the left lung. Innumerable miliary nodules distributed throughout both lungs. Small to moderate amount of intra-abdominal fluid.	Cocci IgG/M positive, CF titer 1:32	No	No
36	Fatigue, Anorexia, Abdominal Distention	CT scan of the abdomen had raised the possibility of peritoneal carcinomatosis.<br><br>CT scan of the chest showed a solitary, 5-mm nodule on the left upper lobe.	Positive IgM/IgG by EIA, CF not done	NA	NA
37	Red spots on skin, fatigue	CT chest, abdomen showed 17 mm of nodule in the anterior subpleural aspect of the right upper lobe with peripheral incomplete surrounding calcifications. Several additional sub centimeter nodules.<br><br>The right kidney with mild diffuse thinning of the cortex.	Cocci IgG by EIA was positive; IgM by EIA was negative.<br><br>ID IgG and IgM were both positive.<br>CF 1:64.	Yes	NA

Abbreviations;

CF, complement fixation

CT, computed tomography

EIA, enzyme immunoassay

ID, immunodiffusion

PET, position emission tomography

US, ultrasound

Supplemental Table S3. Peritoneal fluid analyses for patients with available data (n=15)

Patient Number	Ascitic fluid analysis
Patient 7	10,013 TNC<br>11% eosinophils<br>20% PMNs
Patient 8	489 TNC<br>20% neutrophils<br>50% lymphocytes<br>30% monocytes<br>0% eosinophils
Patient 10	1950 TNC<br>3% neutrophils<br>58% lymphocytes<br>22% monocytes<br>13% eosinophils
Patient 11	3582 TNC<br>2% neutrophils<br>76% lymphocytes<br>14% monocytes<br>6% eosinophils.
Patient 13	3942 TNC<br>15% neutrophils<br>74% lymphocytes<br>8% monocytes<br>3% eosinophils
Patient 17	4724 TNC<br>23% neutrophils<br>46% lymphocytes<br>27% monocytes<br>0% eosinophils
Patient 20	3151 TNC<br>4% neutrophils<br>53% lymphocytes<br>6% eosinophils
Patient 21	9092 TNC<br>38% neutrophils<br>50% lymphocytes<br>11% monocytes<br>1% eosinophils.
Patient 22	6195 TNC<br>24 % neutrophils<br>58% lymphocytes<br>70% monocytes<br>1% eosinophil
Patient 24	4752 TNC<br>1% neutrophils

	46% lymphocytes<br>6% monocytes<br>37% eosinophils.
Patient 25	5931 TNC<br>36% Neutrophils<br>7% lymphocytes<br>46% monocytes<br>11% eosinophils.
Patient 31	2780 TNC<br>12% eosinophils
Patient 32	447 TNC<br>20% Neutrophils<br>80% Monocytes
Patient 36	18 TNC<br>5% neutrophils<br>5% lymphocytes
Patient 37	6900 TNC<br>72% eosinophil

Abbreviations:

TNC, total nucleated cells

Supplemental table S4 – antifungal therapy, duration of therapy.

Patient	Antifungal therapy used and dosage	duration of therapy (in months)	duration of therapy by 12/31/2025 (months)
1	Fluconazole 400 mg QD	Indefinite	110
2	Fluconazole 400-800 mg QD	30	NA
3	Fluconazole 200-400 mg QD	Indefinite	91
4	Fluconazole 600 mg QD, Itraconazole 200 mg BID	Unknown	NA
5	Fluconazole 400-800 mg QD	132	NA
6	Fluconazole 600-800 mg QD Posaconazole 100-300 mg QD	Remains	109
7	Amphotericin, posaconazole 300 mg QD, fluconazole 600 mg QD	Indefinite	53
8	Fluconazole 400 mg QD, Itraconazole 100-200 mg BID, Isavuconazole standard dosing	30	NA
9	Fluconazole 400 mg QD	30 till death	NA
10	posaconazole 300 mg QD	Remains	49
11	Fluconazole 800 mg QD	Remains	119
12	Fluconazole 400-800 mg QD	Remains	46
13	Isavuconazole 372 mg QD	Remains	45
14	Observation	Zero	NA
15	Fluconazole 400 - 800 mg QD, Itraconazole 200 mg BID, Posaconazole 300 mg QD	Remains	93
16	fluconazole 400 mg QD	6 till death	NA
17	Fluconazole 800 mg QD, Itraconazole 200 mg BID, Voriconazole 200 mg BID, Isavuconazole 372 mg QD	Remains	62

18	Fluconazole 200-600 mg QD, Posaconazole 300 mg QD, Itraconazole 200 mg BID	Remains	59
19	Fluconazole 400 mg QD, Itraconazole 200 mg BID	Remains	13
20	Fluconazole 400 mg QD	Remains	20
21	Fluconazole 800 mg QD, Isavuconazole standard dose	remains	9
22	Fluconazole 400 mg QD, Voriconazole 200 mg BID, Posaconazole	13	NA
23	Fluconazole 400 mg QD	34	NA
24	Fluconazole 800 mg QD	Indefinite, interruption 24 months	86
25	Fluconazole 400 mg QD	3 then restarted treatment in anticipation for SOT	NA
26	Fluconazole 200-400 mg QD	Indefinite till death	142
27	Fluconazole 400 mg QD	13	NA
28	Fluconazole 800 mg QD, Itraconazole 800 mg QD, amphotericin, posaconazole 200 mg BID	Indefinite	42
29	posaconazole 300 mg QD	Remains	129
30	Amphotericin for 2 weeks, Itraconazole 200mg bid for 7 months, changed to 300 mg bid	7 till death	NA
31	Amphotericin for 1 day, then Fluconazole 800 mg per day, then 400 mg per day	12	NA
32	Fluconazole 400 mg per day	66	NA
33	Fluconazole 400 mg per day for 2 years, then 200 mg per day for 1-2	Remains- but patient lost follow up	58

	years and then stopped?		
34	Fluconazole 400 mg per day from 4/2020 till 10/2022, changed to voriconazole	Remains- but patient lost follow up	34
35	Amphotericin & Posaconazole, changed to Fluconazole, voriconazole, then on Isavuconazole	Remains	56
36	Amphotericin for 5 days, then Fluconazole 400 mg per day	Remains at last visit- lost to follow up	5
37	Amphotericin for 10 weeks, then Fluconazole 200 mg QD	Remained till death	117

Abbreviations:

NA, not applicable

QD, daily

Supplemental Table S5 – outcome of patients (n=9) who stopped/completed therapy during follow up period

Patient Number	Antifungal therapy used and dosage	duration of therapy (in months)	Outcome
Patient 3	Fluconazole 400-800 mg QD	30	The patient stopped multiple times due to neuropathy and preference.<br><br>No relapse in 110 month follow-up interval.
Patient 6	Fluconazole 400-800 mg QD	132	No relapse in 48 month follow-up interval.
Patient 9	Fluconazole 400 mg QD, Itraconazole 100-200 mg BID, Isavuconazole standard dosing	30	Asked to stop after 30 months of therapy.<br><br>No relapse in 48-month follow-up interval.
Patient 23	Fluconazole 400 mg QD, Voriconazole 200 mg BID, Posaconazole	13	No relapse in 16-month interval follow-up.
Patient 24	Fluconazole 400 mg QD	34	No relapse in 28-month follow up interval.
Patient 26	Fluconazole 400 mg QD	3 then restarted therapy in anticipation for SOT	No relapse in 12-month follow up interval.<br><br>Decision was made to initiate therapy in anticipation for SOT.
Patient 28	Fluconazole 400 mg QD	13	No follow up with infectious diseases.<br><br>Based on follow up visits with other specialties, no documented relapse in 84-month follow-up interval.
Patient 32	Amphotericin for 1 day, then Fluconazole 800 mg QD, then QD	12	No follow-up with infectious diseases.
Patient 33	Fluconazole 400 mg QD	66	No follow-up with infectious diseases.

Abbreviations:

QD, daily

SOT, solid organ transplantation
